# Bout Length-Specific Physical Activity and Adherence to Physical Activity Recommendations among Japanese Adults

**DOI:** 10.3390/ijerph16111991

**Published:** 2019-06-04

**Authors:** Masaki Machida, Tomoko Takamiya, Noritoshi Fukushima, Yuko Odagiri, Hiroyuki Kikuchi, Shiho Amagasa, Makiko Kitabayashi, Jun Kitayuguchi, Shigeru Inoue

**Affiliations:** 1 Department of Preventive Medicine and Public Health, Tokyo Medical University, 6-1-1 Shinjuku, Shinjuku-ku, Tokyo 160-8402, Japan; machida@tokyo-med.ac.jp (M.M.); fukufuku@tokyo-med.ac.jp (N.F.); odagiri@tokyo-med.ac.jp (Y.O.); kikuchih@tokyo-med.ac.jp (H.K.); amagasa@tokyo-med.ac.jp (S.A.); inoue@tokyo-med.ac.jp (S.I.); 2 Faculty of Health and Nutrition, Yamagata Prefectural Yonezawa University of Nutrition Sciences, 6-15-1 Torimachi, Yonezawa-shi, Yamagata 992-0025, Japan; kitabayashi.makiko@gmail.com; 3 Physical Education and Medicine Research Center UNNAN, 328 Kamochouji, Unnan-shi, Shimane 699-1105, Japan; junk_907@yahoo.co.jp

**Keywords:** physical activity, bouts, recommendations, guidelines, accelerometer, adults, Japanese, epidemiology

## Abstract

We aimed to clarify the patterns of moderate to vigorous physical activity (MVPA) in the Japanese adult population, and the proportion of people meeting the recommendations of the Physical Activity Guidelines (PAG) for Americans, second edition (2nd PAG; ≥150 min/week of total MVPA including bouts of any length) and those meeting the previously recommended PAG (2008-PAG; of ≥150 min/week of total MVPA lasting 10 min or longer [long-bout MVPA]). A total of 204 adults (aged 18 to 64 years) from two workplaces were asked to wear an accelerometer. MVPA was classified by bout length, and the proportion of long-bout MVPA was clarified. The proportion of participants adhering to the 2008-PAG and the 2nd PAG recommendations was calculated. Valid data was obtained from 184 adults. Long-bout MVPA accounted for 13.4% of total MVPA. Our results showed that 12.5% of individuals performed MVPA as recommended by the 2008-PAG whereas 92.4% performed MVPA as recommended by the 2nd PAG. Our results, hence, showed that long-bout MVPA comprised only a small proportion of total MVPA, and the proportion of individuals who satisfied the criteria stated in the guidelines (≥150 min/week) significantly changed by whether or not bout length of MVPA was taken into account.

## 1. Introduction 

Being physically active is associated with many health benefits, such as lower risk of all-cause mortality, lower incidence of cardiovascular diseases and some types of cancers, musculoskeletal health benefits, and mental health benefits [1,2]. The Physical Activity Guidelines for Americans 2008 (2008-PAG) recommended ≥150 min/week of moderate to vigorous physical activity (MVPA) lasting at least 10 min (long-bout MVPA), based on previous studies that mostly investigated long-bout MVPA using a questionnaire [1,3]. However, recent studies have shown the beneficial effects of intermittent MVPA lasting less than 10 min (short-bout MVPA) [4,5,6,7]. Based on these studies, the second edition of the Physical Activity Guidelines for Americans (2nd PAG) was published [8]. The 2nd PAG recommends the same duration (≥150 min/week) of MVPA but of any bout length [8]. Total MVPA per week calculated using this new PAG without taking bout length into consideration may be considerably different from total long-bout MVPA per week. These differences are thought to be attributable to different MVPA patterns; and the proportion of long-bout MVPA among total MVPA. Thus, the bout-length specific MVPA patterns are of interest [9].

Previous studies report that long-bout MVPA accounts for only approximately 10% to 25% of total MVPA in Western countries [9,10,11]. These results suggested that total MVPA time of any bout length comparable to 150 min/week of long-bout MVPA may be considerably longer than 150 min/week. However, there are no studies to date that have confirmed this hypothesis. Furthermore, previous studies analyzed MVPA patterns in a limited number of populations [9,12]. Different cultures may affect MVPA patterns, and thus studies of various populations with different cultures are needed. Regarding the Japanese population, we previously reported that long-bout MVPA accounted for approximately 27% of total MVPA time (46.5 min/day) in older subjects, and the percentage of subjects that met the guidelines varied greatly depending on whether or not bout length was taken into consideration [12]. On the other hand, a study focusing on MVPA patterns in Japanese adults has not been reported to date.

Therefore, in this study we aimed to clarify the patterns of MVPA performed by Japanese adults and the proportion of individuals performing MVPA recommended by the 2008-PAG and the 2nd PAG.

## 2. Materials and Methods

### 2.1. Study Design and Participants

This was a cross-sectional study. The participants were recruited from 2 workplaces. One was a local government office with 671 employees in Unnan city, Shimane prefecture, which is located in a rural part of Japan (area: 553.2 km^2^; population: 39,032). All employees in this workplace were office workers. The other workplace was a motor factory with 152 employees in Chino city, Nagano prefecture, which is located in a mountainous region of Japan (area: 266.6 km^2^; population: 55,673). In this workplace, 53 employees were manual laborers and 99 were office workers. Among the 823 employees, 11 who were on leave (maternal leave, etc.) were excluded from the study. The remaining 812 subjects (men: 606, women: 206) were invited to participate in the study. As a result, 204 subjects agreed to participate in this study (response rate, total: 25.1%; men: 25.2%; women: 24.8%). Data collection took place between September and October 2014 at the factory and in August 2018 at the government office.

### 2.2. Questionnaire Data

Information on age, sex, smoking status (current smoker/former smoker/never smoker), and educational level (years of education) was obtained through the self-reported questionnaire. Body mass index (BMI) (kg/m^2^) was calculated from the self-reported height and weight.

### 2.3. Accelerometry

Physical activity was measured using the tri-axial accelerometer Active style Pro HJA-350IT and HJA-750C (Omron Healthcare Co. Ltd., Japan), with a 60 s epoch length. Metabolic equivalents (METs) determined by Active style Pro have been shown to strongly correlate with METs measured by indirect calorimetry [13].

Participants were instructed to wear the accelerometer on their waist throughout the day, except during sleeping or water activities, for 7 consecutive days. Records taken when wearing the accelerometer for at least 10 hours per day were considered valid records [14], and records were defined as “non-wear” if no acceleration signal was observed for more than 60 consecutive min. Participants with data from at least 4 days were included in the analysis [15].

### 2.4. Statistical Analysis

**Figure 1:**
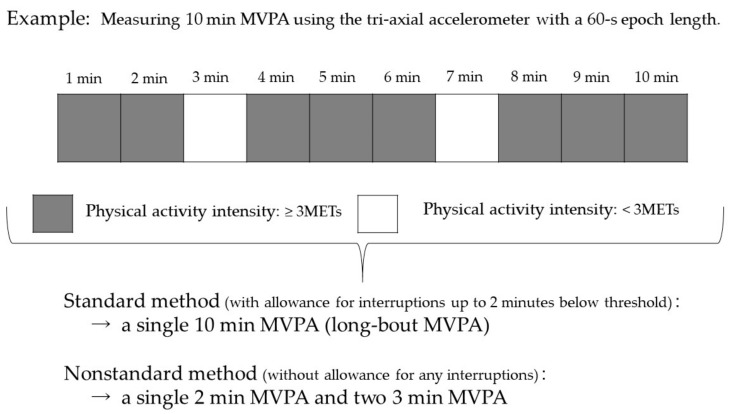
Demonstration of the two definitions of long-bout MVPA (standard method and nonstandard method). MVPA: moderate to vigorous physical activity; standard method: Long-bout MVPA is defined as ≥10 consecutive min with an intensity of 3 METs or higher, with allowance for interruptions of ≤ 2 min below threshold; nonstandard method: Long-bout MVPA is defined as ≥10 consecutive min with an intensity of 3 METs or higher, without allowance for any interruptions.

The accelerometer estimates the intensity of physical activity based on METs. The algorithm for the prediction of METs was established by the Douglas bag method in a controlled laboratory setting [16,17]. Physical activity with an accelerometer intensity of 3 METs or higher was defined as MVPA [18]. Based on a previous study, we categorized MVPA by the length of each bout (1–4 min., 5–9 min., 10–19 min., 20–29 min., and ≥30 min.) [9]. We described the number of MVPA bouts performed in each bout length category, the percentage of MVPA bouts of a particular bout length within the total daily MVPA bouts (total MVPA bouts), total MVPA time by bout length, and the percentage of this MVPA time among total daily MVPA time (total MVPA time) by sex. Two definitions of long-bout MVPA were used in this study, compatible with 2 MVPA recommendations by the 2008-PAG and 2nd PAG (Figure 1). The first is the standard definition used in a previous study [14], defined as 10 or more consecutive min with an intensity of 3 METs or higher, with allowance for interruptions up to 2 min below threshold (standard method, Figure 1), and the second one was without allowance for any interruptions (nonstandard method, Figure 1). Differences between the sexes regarding each activity time and proportion were analyzed using the Mann-Whitney U test.

Total MVPA time per week was calculated by multiplying total MVPA per day by 7. Similarly, long-bout MVPA time per week was calculated by multiplying long-bout MVPA per day by 7. The proportion of participants who engaged in ≥150 min/week of long-bout MVPA, as recommended by both the 2008-PAG and the WHO guidelines on physical activity [3,19] were recorded. Furthermore, the proportion of participants who engaged in ≥150 min/week of MVPA of any bout length, as recommended by the 2nd PAG, and the proportion of those who engaged in ≥300 min/week of MVPA of any bout length, which is another recommendation of the 2nd PAG for additional health benefits [8], were also determined.

For all analyses, *p*-values of less than 0.05 were considered statistically significant. Statistical analyses were performed using IBM SPSS Statistics for Windows, version 24 (IBM Japan, Ltd., Tokyo, Japan).

## 3. Results

### 3.1. Participant Enrolment and Characteristics

Of the 204 subjects surveyed, 20 were excluded for the following reasons: Not meeting the accelerometer wearing time criteria (i.e., wearing for ≥10 h/day for at least 4 days) (*n* = 12), system error (*n* = 1), and age of 65 years or older (*n* = 7). Thus, a total of 184 subjects (133 men; mean age ± standard deviation: 43.4 ± 9.7 years) were included in the present study.

The participants were aged between 18 and 64 years, with the men aged 43.5 ± 10.4 years and women 43.2 ± 7.8 years (Table 1). The percentage of overweight participants (BMI ≥25 kg/m^2^) was 23.3% in men and 9.8% in women.

### 3.2. Patterns of MVPA

**Figure 2:**
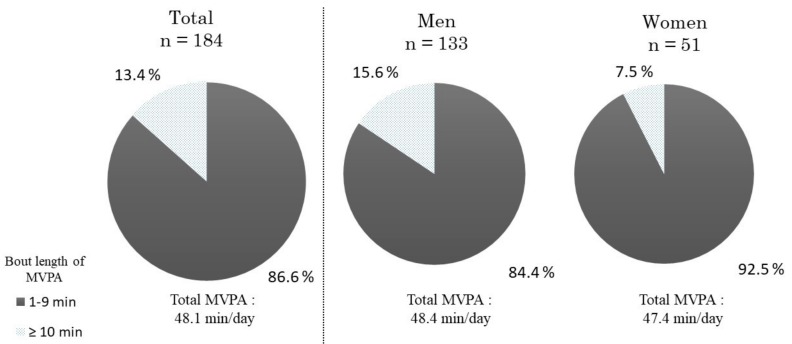
Proportion of bout length-specific moderate to vigorous physical activity (MVPA). Bout length of ≥10 min was MVPA lasting for 10 min or longer, with allowance for interruptions of ≥2 min below threshold.

The total number of MVPA bouts was 27.5 ± 10.0 bouts/day across all participants (men: 26.6 ± 9.9 bouts/day; women: 29.6 ± 10.1 bouts/day) (Table 1). Total MVPA time was 48.1 ± 21.4 min/day (men: 48.4 ± 22.9 min/day; women: 47.4 ± 17.0 min/day). MVPA lasting between 1 to 4 min accounted for 95.3% of total MVPA bouts (men: 94.9%; women: 96.4%) and 80.0% of total MVPA time (men: 78.1%; women: 84.8%) using the nonstandard method (Table 2). Using the standard method, MVPA lasting between 1 to 9 min accounted for 86.6% of the total MVPA time (Figure 2). On the other hand, long-bout MVPA was low at 13.4% (men: 15.6%; women: 7.5%). When comparing the sexes, there was no significant difference in total MVPA time (*p* = 0.934). However, the percentage of long-bout MVPA time among total MVPA time was significantly higher in men than in women (*p* = 0.031).

### 3.3. Adherence to 2008-PAG and 2nd PAG Recommendations

A total of 12.5% of the subjects (men: 17.3%; women: 0.0%) met the 2008-PAG and WHO guideline recommendations on physical activity (Table 1), whereas 92.4% (men: 90.2%; women: 98.0%) met the 2nd PAG recommendations. In addition, the percentage of subjects who engaged in ≥300 min/week of MVPA of any bout length was 53.8% (men: 52.6%; women: 56.9%).

## 4. Discussion

In this study, we showed that Japanese adults aged between 18 and 64 years engaged in MVPA for approximately 48 min a day. Long-bout MVPA, which is recommended by 2008-PAG and by the current WHO guidelines, accounted for only 13.4% (men: 15.6%; women: 7.5%). On the other hand, short-bout MVPA, specifically lasting for 1 to 4 min, accounted for a large part of daily MVPA. From the viewpoint of guideline adherence, 12.5% (men: 17.3%; women: 0.0%) of the participants met the recommendations of the 2008-PAG, whereas 92.4% (men: 90.2%; women: 98.0%) met the recommendations of the 2nd PAG, in which MVPA of any bout length is included.

Luzak et al. reported that long-bout MVPA accounted for 14% of the total MVPA time in German subjects aged between 48 and 68 years [9]. Salvo et al. showed that long-bout MVPA comprised 9.7% of the total MVPA time in Mexican adults aged between 20 and 65 years [11]. Our results generally agree with these findings, which suggest that the proportion of long-bout MVPA is small. In addition, Jefferis et al. reported that short-bout MVPA accounted for 75% of the total MVPA time, meaning that long-bout MVPA accounted for 25% in elderly individuals living in the United Kingdom [10]. We also previously reported that long-bout MVPA accounted for 26.9% of the total MVPA time in elderly Japanese individuals aged 70 years and older [12]. Our previous research and this study are the first studies to describe the pattern of MVPA in an Asian population, taking bout length into consideration. Our results indicated that although the degree varies depending on the characteristics of the target population, short-bout MVPA accounts for a large proportion of MVPA across all populations.

In this study, only 12.5% of the participants met the 2008-PAG and the WHO guidelines, which recommend ≥150 min/week of long-bout MVPA; however, 92.4% of them met the 2nd PAG that recommend ≥150 min/week of total MVPA. Arredondo et al. analyzed Hispanic and Latino adults in the United States and reported that 16.4% of men and 10.6% of women engaged in ≥150 min/week of long-bout MVPA whereas 51.1% of men and 31.3% of women engaged in ≥150 min/week of total MVPA [20]. Jefferis et al. studied older men in the United Kingdom and reported that 16% of them engaged in ≥150 min/week of long-bout MVPA whereas 66% of them engaged in ≥150 min/week of total MVPA [4]. These results combined with our results highlight even more clearly the discrepancy in the percentage of those who meet the guidelines depending on whether the bout length of physical activity is considered, although the degree may vary depending on the target population. Compared with previous studies, the participants of our present study showed a larger discrepancy in the percentage of participants meeting the different guidelines [4,20], and most Japanese adults met 2nd PAG. Previous studies reported that daily long-bout MVPA time was approximately 5 to 10 min, and total MVPA time was approximately 20 to 30 min in Western countries [4,5,6,9,11,20]. On the other hand, total MVPA time was 48 min in the present study, which was longer than previous studies, whereas long-bout MVPA time was 8.3 min, which was similar to previous studies. Our results suggested that the prevalence of adherence to the recommendations may differ greatly depending on whether or not the guidelines take bout length into consideration, particularly in a population in which most individuals accumulate daily MVPA time by short bout MVPA with less long-bout MVPA. Therefore, it may be difficult to identify individuals in need of intervention using the 2nd PAG in a population like the Japanese who accumulate MVPA time mostly by short-bout MVPA, resulting in most of the population meeting the recommendations. 

Furthermore, we compared the pattern of MVPA and the prevalence of adherence to 2008-PAG and 2nd PAG recommendations by sex. Our results showed that there was no significant difference in the total MVPA time between the sexes, whereas long-bout MVPA was longer and the proportion of long-bout MVPA within total daily MVPA was significantly higher in men than in women. Amagasa et al. reported that the average daily short-bout MVPA time is longer in older women than in older men whereas long-bout MVPA time is longer in older men than in older women [21]. Our present study on adults showed comparable results as this previous study. In addition, our result showed that the proportion of subjects who adhered to the 2nd PAG recommendations was higher in women than in men, although the proportion of subjects who adhered to 2008-PAG recommendations was higher in men than in women. In the many previous studies focusing on long-bout MVPA, men have been regarded as being more active than women, regardless of age [22,23]. However, this tendency might just be owing to short-bout MVPA being overlooked. The results of our present study suggest that it may not necessarily be the case that men are more active than women, if short-bout MVPA is taken into consideration to evaluate physical activity.

This is the first study clarifying the patterns of MVPA focusing on bout length in Asian adults using accelerometry. Our results indicate that the proportion of the active adult population differs greatly depending on which guidelines are used, i.e., whether long-bout MVPA is taken into consideration or not. Therefore, care must be taken when identifying the target population in need of intervention regarding insufficient physical activity.

There are some limitations that should be considered in our study. Firstly, the tri-axial accelerometer (Active style Pro) used in this study was a different accelerometer from those used in previous studies in Western countries [9,10,11]. Active style Pro has shown great validity and is widely used in Japan [13,16], and it was reported to accurately measure energy expenditure in free-living conditions [13]. However, algorithms for calculating the physical activity index are different for each accelerometer [16,24,25]. Thus, it is difficult to compare this study with studies in which different tri-axial accelerometers were used. Secondly, selection bias should be considered. The response rate was low, and some subjects were excluded because of insufficient wear time. Therefore, the participants in this study may be more active than the actual targeted population. Thirdly, the sample size was relatively small. In addition, the participants in this study were only workers from two different workplaces, and the number of women included in the study was small. Thus, our results may not be generalized to all professions or nonworkers. Further research using a larger sample size and subjects from various professions, including nonworkers, is required.

## 5. Conclusions

Long-bout MVPA accounted for approximately 13% of the daily total MVPA time in Japanese adults. Short-bout MVPA, specifically lasting for 1 to 4 min, made up a large part of the daily total MVPA. The percentage of subjects who met the guidelines varied greatly depending on whether or not bout length was taken into consideration.

## Figures and Tables

**Table 1:** Characteristics of the participants.

Background Information	Total	Men	Women
	(*n* = 184)	(*n* = 133)	(*n* = 51)
Age (years) (mean ± SD)	43.4 ± 9.7	43.5 ± 10.4	43.2 ± 7.8
Workplace			
Government office (*n* [%])	88 (47.8%)	48 (36.1%)	40 (78.4%)
Factory (*n* [%])	96 (52.2%)	85 (63.9%)	11 (21.6%)
Occupation			
Office worker	138 (75.0%)	94 (70.7%)	44 (86.3%)
Manual laborer	46 (25.0%)	39 (29.3%)	7 (13.7%)
Body mass index			
<25.0 kg/m^2^ (*n* [%])	148 (80.4%)	102 (76.7%)	46 (90.2%)
≥25.0 kg/m^2^ (*n* [%])	36 (19.6%)	31 (23.3%)	5 (9.8%)
Smoking status			
Current smoker (*n* [%])	59 (32.1%)	50 (37.6%)	9 (17.6%)
Former smoker (*n* [%])	33 (17.9%)	31 (23.3%)	2 (3.9%)
Never smoker (*n* [%])	92 (50.0%)	52 (39.1%)	40 (78.4%)
Educational level			
≥13 years (*n* [%])	121 (65.8%)	80 (60.2%)	41 (80.4%)
<13 years (*n* [%])	63 (34.2%)	53 (39.8%)	10 (19.6%)
MVPA			
Total number of MVPA bouts (bouts/day) (mean [SD])	27.5 (10.0)	26.6 (9.9)	29.6 (10.1)
Total MVPA ^1^ time (min/day) (mean [SD])	48.1 (21.4)	48.4 (22.9)	47.4 (17.0)
Long-bout MVPA ^2^ ≥150 min per week (*n* [%])	23 (12.5%)	23 (17.3%)	0 (0.0%)
Total MVPA ≥150 min per week (*n* [%])	170 (92.4%)	120 (90.2%)	50 (98.0%)
Total MVPA ≥300 min per week (*n* [%])	99 (53.8%)	70 (52.6%)	29 (56.9%)

SD: Standard deviation; MVPA: Moderate to vigorous physical activity. ^1^ moderate to vigorous physical activity of any bout length >1 min. ^2^ ≥10 consecutive min with intensity of 3 METs or higher, with allowance for interruptions of ≤2 min below threshold.

**Table 2:** Bout length-specific moderate to vigorous physical activity patterns.

Bout Length (min)	Number of MVPA Bouts (Times/Day)			Percentage of MVPA Bouts (%)			MVPA Time (Min/Day)			Percentage of MVPA Time (%)		
	Total	Men	Women	Total	Men	Women	Total	Men	Women	Total	Men	Women
	*n* = 184	*n* = 133	*n* = 51	*n* = 184	*n* = 133	*n* = 51	*n* = 184	*n* = 133	*n* = 51	*n* = 184	*n* = 133	*n* = 51
**Nonstandard Method ^1^** (without allowance for any interruptions)												
1–4	26.2 (9.6)	25.3 (9.5)	28.6 (9.8)	95.3 (4.0)	94.9 (4.3)	96.4 (2.6)	37.0 (14.7)	35.9 (14.7)	40.0 (14.6)	80.0 (15.7)	78.1 (17.2)	84.8 (9.6)
5–9	1.0 (0.8)	1.0 (0.9)	1.0 (0.7)	3.7 (3.3)	3.9 (3.5)	3.3 (2.5)	6.1 (5.4)	6.2 (5.8)	5.8 (4.2)	12.2 (9.7)	12.3 (10.1)	12.1 (8.7)
≥10	0.3 (0.5)	0.3 (0.5)	0.1 (0.1)	1.0 (1.8)	1.2 (2.0)	0.3 (0.5)	5.0 (10.1)	6.3 (11.5)	1.5 (2.3)	7.8 (13.1)	9.6 (14.7)	3.2 (5.2)
10–19	0.2 (0.4)	0.2 (0.4)	0.1 (0.1)	0.7 (1.5)	0.8 (1.7)	0.3 (0.5)	2.4 (4.8)	2.9 (5.5)	0.9 (1.5)	4.1 (7.8)	5.0 (8.9)	2.0 (3.4)
20–29	0.0 (0.1)	0.0 (0.1)	0.0 (0.0)	0.1 (0.4)	0.1 (0.5)	0.0 (0.1)	0.8 (2.8)	1.0 (3.2)	0.2 (0.8)	1.1 (3.7)	1.4 (4.2)	0.4 (1.8)
≥30	0.0 (0.2)	0.1 (0.2)	0.0 (0.0)	0.2 (0.6)	0.2 (0.7)	0.0 (0.1)	1.8 (7.3)	2.4 (8.5)	0.3 (1.7)	2.5 (9.0)	3.2 (10.2)	0.8 (4.0)
Total	27.5 (10.0)	26.6 (9.9)	29.6 (10.1)	100	100	100	48.1 (21.4)	48.4 (22.9)	47.4 (17.0)	100	100	100
**Standard Method ^2^** (with allowance for interruptions of up to 2 min below threshold)												
≥10	0.5 (0.6)	0.5 (0.7)	0.3 (0.3)	1.6 (2.1)	1.9 (2.4)	0.9 (0.8)	8.3 (12.8)	10.0 (14.5)	3.7 (4.1)	13.4 (16.1)	15.6 (17.8)	7.5 (8.1)
10–19	0.4 (0.5)	0.4 (0.6)	0.2 (0.3)	1.3 (1.7)	1.4 (1.9)	0.8 (0.8)	4.8 (7.0)	5.6 (7.8)	2.8 (3.4)	8.4 (10.3)	9.4 (11.3)	5.6 (6.4)
20–29	0.0 (0.1)	0.1 (0.2)	0.0 (0.1)	0.2 (0.5)	0.2 (0.6)	0.1 (0.2)	1.1 (3.4)	1.3 (3.8)	0.4 (1.3)	1.7 (4.6)	2.0 (5.0)	0.9 (3.4)
≥30	0.1 (0.2)	0.1 (0.2)	0.0 (0.0)	0.2 (0.7	0.3 (0.8)	0.0 (0.2)	2.4 (8.3)	3.1 (9.6)	0.5 (2.0)	3.3 (10.4)	4.2 (11.8)	1.0 (4.6)

Values are shown as mean (standard deviation); MVPA: Moderate to vigorous physical activity. ^1^ Long-bout MVPA is defined as ≥10 consecutive min with intensity of 3 METs or higher, without allowance for any interruptions. ^2^ Long-bout MVPA is defined as ≥10 consecutive min with intensity of 3 METs or higher, with allowance for interruptions of ≥2 min below threshold.
